# Improved Reference Genome Sequence of *Coccidioides immitis* Strain WA_211, Isolated in Washington State

**DOI:** 10.1128/MRA.00149-19

**Published:** 2019-08-15

**Authors:** Marcus de Melo Teixeira, Bridget Marie Barker, Jason E. Stajich

**Affiliations:** a Faculty of Medicine, University of Brasília, Brasília, Federal District, Brazil; b The Pathogen and Microbiome Institute, Northern Arizona University, Flagstaff, Arizona, USA; c Department of Microbiology & Plant Pathology and Institute for Integrative Genome Biology, University of California, Riverside, Riverside, California, USA; Broad Institute

## Abstract

Coccidioides fungi are widely distributed in the American continents, with an expanding western range documented by a recently discovered cryptic population of Coccidioides immitis in Washington State. The assembled and annotated reference genome sequence of the soil-derived C. immitis strain WA_211 will support population and functional genomics studies.

## ANNOUNCEMENT

Coccidioides immitis and Coccidioides posadasii are fungal species found in desert-like areas of the American continents (1) and are the causative agents of coccidioidomycosis or “valley fever.” While *C. posadasii* is broadly distributed, *C. immitis* is restricted to southern California and northern Mexico. Infections due to *C. immitis* in California are increasing and reached alarming rates in 2017 (2). The disease range appears to be expanding, with uncommon autochthonous infections reported in Washington State (3). Washington *C. immitis* isolates from the soil and clinic are reciprocally monophyletic within *C. immitis* (4, 5) and rarely hybridize with *C. posadasii* (6). As most assembled genomes of *C. immitis* are patient-derived isolates, we annotated and assembled the genome of the soil-derived strain WA_211, as it represents a unique emerging lineage of *C. immitis*, for comparative, population, and functional genomics research.

The WA_211 strain was cultured from soil on yeast extract medium at 37°C for 5 days (5), and its DNA was isolated after growth on Sabouraud’s medium using the DNeasy blood and tissue kit (Qiagen, Hilden, Germany). The sequencing library was prepared with a Kapa Biosystems (Woburn, MA) kit (catalog number kk8201) and sequenced with an Illumina MiSeq platform (2 × 300 bp) (4, 5). The 2.8 million read pairs (1.7 Gbp) were obtained from the SRA and processed with shovill v.1.0.4 (https://github.com/tseemann/shovill) using “–minlen 500 –trim” and shovill defaults for downstream tools. The shovill pipeline trimmed reads for adaptors and low quality using Trimmomatic v.0.39 (7), corrected bases using Lighter v.1.1.2 (8), and merged overlapping read pairs using FLASH v.1.2.11 (9) to produce 1.6 million merged reads and 1 million unmerged read pairs. Contigs were assembled with SPAdes v.3.10.1 (10), polished with one round of Pilon v.1.22 (11), and cleaned of vector sequence and redundant contigs by AAFTF v.0.2.1 (12), using default parameters. The 297 contigs were scaffolded to the *C. immitis* RS genome (GenBank accession number AAEC00000000) (13) with RagOO v.1.1 (14), with default parameters, to produce a 27.4-Mb assembly of 62 scaffolds (*N*_50_, 3.79 Mb; longest scaffold length, 8.24 Mb; G+C content, 46.4%). This scaffolding assumes the colinearity of WA_211 and RS, but no breakpoints were observed within contigs in a comparison of a dotplot by D-GENIES (15).

The genome contains 15% repetitive sequences masked by RepeatMasker v.open-4.0.7 using a *Coccidioides* repeat element library (16). Genes were predicted in the masked genome with Funannotate v.1.5.2 (17). Gene prediction parameters were generated by “funannotate train” using alignments of *C. immitis* spherule and hyphal RNA sequencing (RNA-seq) (18). Gene prediction (“funannotate predict”) was performed running *ab initio* predictors Augustus (19), SNAP v.2013-11-29 (20), CodingQuarry v.2.0 (21), and GeneMark-ES v.4.33 (22) using exon hints from spliced alignments of transcripts, Onygenales proteins (13, 23, 24), and the Swiss-Prot database (25). Consensus gene models were generated by Funannotate running EVidenceModeler v.1.1.1 (26). Funannotate assigned putative gene products by searches to the Swiss-Prot, InterPro (27), eggNOG (28), MEROPS (29), and dbCAN (30) databases, using default parameters. A total of 7,815 protein-coding gene models were predicted, of which 5,477 had InterPro domains. Twenty-one secondary metabolite clusters were predicted by antiSMASH 4.0 (31), comprising 8 polyketide synthases (PKS), 4 nonribosomal peptide synthetases (NRPS), 2 hybrid PKS-NRPS, 1 indole-NRPS, 3 terpene, and 4 “other” type clusters. The annotation and assembly pipeline steps, full parameters, and log files are archived in the GitHub repository (32).

### Data availability.

This whole-genome shotgun project has been deposited at DDBJ/ENA/GenBank under the accession number RHJW00000000. The version described in this paper is version RHJW02000000. The genomic sequence reads used in this assembly were previously deposited under SRA project accession number SRR1292227, and the RNA-seq reads used had been deposited under BioProject number PRJNA169242 and SRA accession number SRP013923.
